# Essential Role of the Innate Immune Adaptor RIP2 in the Response to Otitis Media

**DOI:** 10.3389/fgene.2022.893085

**Published:** 2022-07-12

**Authors:** Arwa Kurabi, Jasmine Lee, Kwang Pak, Anke Leichtle, Allen F Ryan

**Affiliations:** ^1^ Department of Surgery, Division of Otolaryngology, University of California, San Diego, San Diego, CA, United States; ^2^ Division of Biological Sciences, University of California, San Diego, San Diego, CA, United States; ^3^ Department of Otolaryngology, University of Lübeck, Lübeck, Germany; ^4^ San Diego Veterans Administration Healthcare System, La Jolla, CA, United States

**Keywords:** receptor interacting protein 2 (RIP2), inflammation, pro-inflammatory cytokines, ear infections, TLR—toll-like receptor, NLR (NOD-like receptors), mucosal immmunity

## Abstract

Intracellular nucleotide binding and oligomerization domain (NOD) and Toll-like (TLR) receptors have emerged as pivotal sensors of infection. Both Nod1 and Nod2 contain a caspase activation and recruitment domain (CARD) that interacts with the adaptor protein RIP2 (receptor-interaction protein-2). This leads to ubiquitination of RIP2 and in turn to the activation of NFκB and MAPK transcription factors, to command the host defensive response against pathogenic infections. RIP2 is also activated by TLRs 2 and 4, although the mechanism of this activation is less. The role of RIP2 in otitis media (OM) pathogenesis has yet to be examined. Herein, we used *in vivo* animal models including C57BL/6 wild-type (WT) and RIP2^−/−^ knockout mice inoculated in the middle ear (ME) with non-typeable *Haemophilus influenzae* (NTHi), a common human OM pathogen, to evaluate the expression of RIP2 and its signaling genes at the cellular level to determine the role of RIP2 in OM pathogenesis and recovery. The *Nod1*, *Nod2*, and *Ripk2* genes are minimally expressed in the normal ME. However, they are strongly upregulated during acute OM, as are many genes related to RIP2 signaling. However, while signaling genes were expressed by various ME cell types, only mucosal epithelial and stromal cells expressed the NODs, RIP2, and signaling genes required for the activation of the host defensive response. Whereas WT mice clear ME bacteria and recover from OM within 5 days after infection, RIP2-deficient mice show persistent ME bacterial carriage and inflammation to at least 15 days. This includes significantly prolonged mucosal hyperplasia and ME leukocytic infiltration. Recruitment of macrophages is also delayed in comparison to WT mice. Thus, RIP2 is required to elicit a robust innate immune response that promotes bacterial clearance and increases host innate resistance. The results also identify the structural cells of the ME mucosa, as opposed to leukocytes, as the primary sites of NOD/RIP2 activity in the infected ME.

## Introduction

Otitis media (OM) is one of the most common conditions seen by pediatricians. (Casselbrant et al., 1993). Acute otitis media (AOM) and chronic OM (COM) account for more physician appointments, antibiotic prescriptions, and surgical procedures than any other childhood disorder (Klein, 2000). It is estimated that disease diagnosis, treatment and associated costs exceed $5 billion per year in the United States (Elden and Coyte, 1998). However, OM is a much more serious disease in many developing countries. The WHO estimates that undertreated OM leads to at least 28,000 annual childhood deaths due to intracranial infections and is the cause of one half of the world’s burden of handicapping hearing loss (WHO, 2004; WHO, 2020 GBD). Treatments for OM have changed little in the past 50 years. Improved understanding of the OM pathogenesis and recovery is important for improving treatment options and outcomes.

The most common pathogens known to cause OM are *Streptococcus pneumoniae*, non-typeable *Haemophilus influenzae* (NTHi), and *Moraxella catarrhalis* (Kaur et al., 2017). The combination of an increase in bacterial presence in the nasopharynx along with a decrease in activity of cilia in the Eustachian tube (ET) is believed to lead to the development of a bacterial infection in the middle ear (ME) (Park et al., 1993). Sensing of these cellular pathogens triggers the host innate immune system to resist infection as a defense strategy (Si-Tahar et al., 2009). Phagocytes (neutrophils and macrophages) react to signals sent out by natural killer (NK) and other cells and are responsible for the engulfment of the detected pathogen. Neutrophils comprise the majority of the phagocytes present and are the first to influx into the ME cavity. Macrophages tend to arrive later and engulf more pathogens for a longer period of time. Cytokines such as interleukins help to regulate macrophage activity and control the inflammatory response (Plüddemann et al., 2011). The ME mucosa also undergoes significant pathological transformation as it grows rapidly from a simple squamous monolayer of epithelium with minimal stroma into this structured hyperplasic pattern of respiratory epithelium with ciliated, goblet and secretory cells that secrete mucus and ME effusions (Ryan et al., 2020).

Although clearing pathogens is important in resolving the infection, it is only the first step in the recovery process. Normal tissue structure must be regained through the clearance of inflammatory leukocytes through apoptosis at the infection site as well as the prevention of more phagocytes from entering the ME cavity through the mucosal layer. Cytokines such as anti-inflammatory and growth factors must be released to start the process of healing (Serhan and Savill, 2005). Inflammation must be negatively and tightly regulated to reduce tissue damage. Repair and recovery must also be actively promoted. This process must not only be effective but also restricted in its response. Otherwise, chronic inflammation may occur due to failure in resolving infection (Lawrence and Gilroy, 2007).

AOM in a non-OM-prone child resolves within a few days, even without antibiotic treatment (Rosenfeld and Kay, 2003). This is too soon for the elaboration of effective adaptive immunity, which implies that the normal ME response to infection is mediated by innate immunity, which does not require prior sensitization. Innate detection of microbial infection occurs *via* patter recognition receptors (PRRs) that recognize microbial molecules. The most notable PRRs receptors include the Toll-like receptors (TLRs) and NOD-like receptors (NLRs) (Kumar et al., 2011). The role of several TLR and NLR PRRs in OM pathogenesis and recovery has been characterized (e.g., Hernandez et al., 2008; Leichtle et al., 2009; Leichtle et al., 2012; Lee et al., 2019).

**FIGURE 1:**
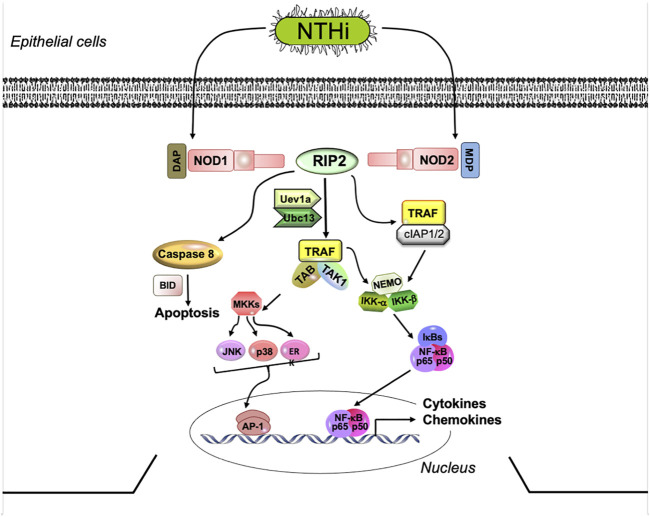
NOD signaling *via* the RIP2 adaptor. NOD-mediated RIP2 signaling strongly stimulates the production of pro-inflammatory cytokines, *via* NFκB and MAPK activation. Several parallel pathways can lead from RIP2 to inflammatory gene expression. NOD, nucleotide-binding oligomerization domain; RIP2, receptor interacting protein kinase 2; IRAK1, interleukin-1 receptor-associated kinase 1; TRAF2,5,6, tumor necrosis factor receptor associated factors 2, 5, and 6; TAK1, mitogen-activated protein kinase 7 (TAK1); NFκB, nuclear factor of kappa light polypeptide gene enhancer in B-cells; p50 NFκB subunit 1; p65, NFκB subunit 3,TNFα, tumor necrosis factor alpha; IL, interleukin.

The NLRs include NOD1 and NOD2, the first members to be identified. These intracellular receptors detect bacterial peptidoglycan (PGN) fragments, including highly conserved muropeptides (Bourhis and Werts, 2007). They are composed of a C-terminal LRR domain, a central nucleotide binding and oligomerization domain, and an N-terminal caspase activation and recruitment domain (CARD). Once activated by bacterial PGNs (Figure 1), NOD1 and NOD2 oligomerize and recruit RIP2 through a homotypic CARD-CARD interaction to trigger RIP2 ubiquitination and polymerization. Polymerized RIP2 acts *via* TRAF1, TRAF5, TRAF6, and/or IAPs to stimulate pro-inflammatory cytokines release *via* NFκB (McCarthy et al., 1998; Reinke et al., 2020) and also *via* intermediate kinases to activate MAPKs including JNK and p38 (Homer et al.,. 2012). RIP2 can also play a role in TLR signaling, since ligand binding to TLR2 and TLR4 has been shown to activate RIP2 through an unclear mechanism (Kobayashi et al., 2002; Lu et al., 2005; Dorsch et al., 2006; Usluoglu et al., 2007), although TLR involvement has been questioned (Park et al., 2007; Hall et al., 2008). In addition, RIP2 has been shown to mediate cell death *via* caspase nine activation (da Silva Correia et al., 2007). Through its ability to initiate and sustain inflammatory responses, RIP2 is important defense molecule in pathogen elimination and infection resolution. However, its involvement in OM has yet to be examined. *In vivo*, RIP2-deficient mice displayed reduced inflammatory cells infiltration and disease pathogenesis in a murine model for multiple sclerosis (Shaw et al., 2011). In mammals, the NOD/RIP2 signaling axis appears conserved and plays a role in innate immune signaling and autophagy (Strober et al., 2006; Heim et al., 2019; Chuphal et al., 2022).

The present study was designed to assess the role of RIP2 in OM induced by NTHi, to identify its involvement in pathogenesis and resolution. Since lack of RIP2 disables the innate immune activity of NOD1 and NOD2, and can reduce the effects of TLR activation, we hypothesized that RIP2 deletion would reduce inflammatory pathogenesis but would also produce a more severe ME infection than observed in WTs or with the deletion of individual PRRs.

## Materials and Methods

### Animals

Single-cell RNA-Seq studies were conducted using 60–90 day old C57BL/6 mice while those for gene array analysis were C57BL/6:CB F1 hybrids (Jackson Labs, Bar Harbor, ME, United States). The RIP2^−/−^ mice on a C57BL/6 background were crossed 10 times to produce the homozygous line. These were generated and provided by Ulevitch and colleagues (Pan et al., 2007). Age-matched C57BL/6 mice were purchased from Jackson Laboratories (Bar Harbor, ME) and used as the WT. All experiments were approved by the Institutional Animal Care and Use Committee of the Veteran Affairs Medical Center (San Diego, CA) and performed according to National Institutes of Health guidelines for the care and use of laboratory animals.

### Bacteria

Non-typeable *Haemophilus influenzae* strain 3655 (biotype II, NTHi) was provided by Dr. Asa Melhus (Lund University) after isolation from the ME of a patient who had OM (Melhus and Ryan, 2003). The strain is validated by genomic DNA sequencing. The culture was streaked onto a chocolate agar plate and placed in a 37°C incubator overnight. Two colonies were then selected and grown in 25 ml of brain heart infusion (BHI) media added with 1 ml of Fildes enrichment (BD Diagnostic Systems). The next day, the bacteria were spun down at 7,000 rpm for 10 min and the pellet resuspended in fresh BHI media. To induce an inflammatory OM response, a final concentration of 10^4^—10^5^ bacteria/ml inoculated into the rodent ME.

### Surgery

RIP2^−/−^ and C57BL/6 mice were divided into groups of 6 mice for each experimental time point (3 each for histopathology and bacterial culture). The mice were deeply anesthetized with an intraperitoneal injection of rodent cocktail (13.3 mg/ml ketamine hydrochloride, 1.3 mg/ml xylazine, 0.25 mg/ml acepromazine). 0.1–0.2 ml of this mixture was given per 25–30 g body weight of the mouse (i.p.). A midline incision was made on the neck by a ventral approach. Soft tissue dissection was done to bilaterally expose both the ME bullae. A hole was carefully drilled with a 20-gauge needle, in which 5 ul of NTHi inoculum was injected into the cavity of the ME. A sterile cotton swab was used to remove excess fluid and the wound was closed through stapling of the skin incision. Lactated Ringer’s solution and buprenorphine was given postoperatively to the animals through a subcutaneous injection. The controls used were uninoculated mice (time—0 day).

### Gene Array

Gene arrays were used to provide quantitative information on gene expression levels within the ME, since mRNA is extracted in a uniform manner from all cells in a tissue. Forty mice per time point were inoculated in the ME bilaterally with NTHi. Uninoculated animals served as controls. Mucosal tissue and exudate was harvested from 20 mice at each of the following intervals: 0 h (0h, no treatment), 3, 6, 24 h, 2 days (2 days), 3, 5ays, and 7 days after inoculation, and pooled. The tissue was homogenized in TRIzol (Life Technologies, Carlsbad, CA) and total RNA extracted, reverse transcribed and transcribed *in vitro* to generate biotinylated cRNA probes that were hybridized to 2 Affymetrix MU430 2.0 microarrays. This procedure was duplicated for each time point to obtain a second, independent biological replicate. Thus each data point represents 2 separate samples consisting of 20 mice each, and 4 Affymetrix arrays. Specific genes were assessed at individual time points, after Bonferroni correction for multiple tests, using Genespring GX 7.3 (Agilent Technologies, Santa Clara, CA). Additional details of methods can be found in Hernandez et al. (2015).

### Single-Cell RNA-Seq

We used single-cell RNA-Seq to provide precise, cell-level data because gene arrays generated from bulk tissue cannot identify which cells are expressing a given gene. However, because the isolation of different cell types will vary in efficiency depending upon fragility and strength of bonding to other cells, this method is less able to determine overall ME levels of gene expression. The expression of RIP2-related genes in individual cell types of the ME was assessed in an existing murine dataset of normal MEs (Ryan et al., 2020), and in MEs inoculated 6 h earlier with NTHi. 6 h was chosen based on gene array data since *Ripk2* expression was strongly upregulated at this time after NTHi inoculation. The data was obtained from three independent groups made of six normal or six infected mice. Subjects were sacrificed and the ME tissue and luminal contents harvested from the 12 MEs of each group. The pooled tissue was digested in thermolysin (0.5 mg/ml, Sigma-Aldrich, #T7902) followed by FACSMax cell dissociation solution (Genlantis, #T200100), and triturated into three single-cell preparations. Dissociated cells were diluted to 700 cells/μl. Three replicate groups were performed to obtain independent biological samples. Single-cell libraries were prepared using the Chromium Controller (10X Genomics, Pleasanton, CA) per manufacturers’ instructions. The libraries were sequenced with Illumina HiSeq 2500 (Illumina, San Diego), yielding approximately 200 million reads per sample. Individually bar-coded reads were demultiplexed using Cellranger 2.0.2 (10X Genomics) and mkfastq in conjunction with bcl2fastq 2.17.1.14 (Illumina) and aligned to the murine reference genome mm10 (Ensembl 93) provided by 10X Genomics. Single-cell libraries were filtered for quality, subjected to principal component analysis (PCA) clustering the three independent samples, and identified based on well-recognized marker genes (Ryan et al., 2020). For the three samples from normal MEs, the average number of analyzed cells/sample was 2,257, and the number of genes detected/sample averaged 17,322. For the three samples at 6 h, the number of cells/sample averaged 2,407 and genes detected averaged 16,795. A count-based quality control filter was used to exclude dead/damaged cells as described previously (Ryan et al., 2020). Identification of cells in each cluster was based on the following marker genes: epithelial cells, *Epcam and Krt18*; ciliated epithelial, *Hydin*; basal epithelial, *Krt5*; stromal cells, *Col1a2*; vascular endothelial cells, *Egfl7* and *Flt4*; lymphatic endothelial cells *Egfl7* and *Flt1*; pericytes, *Rgs5*; monocytes, *Csf1r*; lymphocytes, *Ptprcap*; and melanocytes, *Mlana*. After 6 h infection, PMNs were identified by expression of *Il1f9* and *Stfa2l1* and RBCs by *Hba-a1* hemoglobin gene expression. Log-normalized violin plots were used to evaluate gene related to RIP2 expression levels by each cluster and for optimal comparison across the cluster cell population. Marker genes employed for normal ME and other additional details of methods are available in our previous publication on normal ME scRNA-Seq (Ryan et al., 2020), in which no related genes were reported.

### Histology

The mice used were sacrificed under general anesthesia by intracardiac perfusion. PBS was first injected, followed by 4% paraformaldehyde (PFA). Time points were collected at 0, 6, and 12 h after inoculation, and 1,2,3,5,7,10,14, and 21 days after inoculation. In the mouse OM model, the initial days after bacterial inoculation are very dynamic as the mucosa begins to develop hyperplasia around 1 day after inoculation and by 2 days the mucosal thickness peaks. Tissue recovery occurs over several days but at a slower time scale. The 0 h time point was collected from untreated ears and used as the baseline. The ME bulla was then dissected from the cranium and placed into a 4% PFA solution overnight. They were then transferred to 8% EDTA and 4% PFA solution and decalcified for 14 days. The bullae were embedded into paraffin, sectioned at 7 μm and the sections stained with hematoxylin-eosin. The same region from the largest area of the ME cavity was then digitally recorded. Mucosal thickness was measured in 6 regions of the ME. The measurements were taken at regions where the underlying bone was relatively flat, because at the sharp bends of the ME bulla the stroma is thick. The Eustachian tube orifice region was avoided since the mucosa there is naturally heavily ciliated and thick. Mucosal thickness was analyzed by computer-averaging the thickness of the epithelium and subepithelium stroma at six standardized ME locations. Using image analysis software, the percent area of the ME lumen occupied by inflammatory cells as then measured. Finally, the numbers of neutrophils and macrophages were then counted in five sites within the area of ME cellular infiltrate at × 400 magnification, to determine the relative proportions of these two dominant cell types. All histological measures were performed independently by 2 observers and averaged (Ebmeyer et al., 2005).

### Bacterial Clearance

The ME was opened and a sample from the lumen was obtained using a 1 ul loop which was streaked onto a chocolate agar plate. Each loop was streaked onto 4 quadrants of the plate. The plates were then incubated for 24 h. Verification of NTHi was done through gram-staining the colony forming units and negative cultures that were grown on both blood agar and chocolate agar plates. A scoring system was used to categorize the degree of colonization in order to analyze the colony forming units obtained from the ME lumen cultures (colony score or CS). 0 indicated no colony-forming units on the plate, 1 indicated colony-forming units in 1 quadrant, 2 indicated colony-forming units in 2 quadrants, 3 indicated colony-forming units in 3 quadrants, and 4 indicated colony-forming units in all 4 quadrants on the plate (Hernandez et al., 2008). In addition, all colonies on the plate were totaled to obtain CFUs/ml.

### Macrophage Function

Primary peritoneal macrophages were obtained from six WT mice and six Rip2^−/−^ mice by i. p. injection of 3 ml 4% thioglycolate media. Cells were harvested 3 days later by peritoneal lavage with cold RPMI 1640, containing 10% FBS, 50 U/ml penicillin, and 50 Ig/ml streptomycin and ß-mercaptoethanol, washed with media, counted and seeded into 48-well plates at 5 × 10^5^ cells per well. NTHi were added at 5 × 10^7^ per well. The plates were centrifuged at 100 g for 5 min to enhance NTHi/macrophage contact and then incubated for 1 h at 37°C. Extracellular bacteria were then removed by washing with DMEM, and gentamicin was added at 50 mg/ml to kill any remaining extracellular bacteria. To assess NTHi phagocytosis, macrophages were then immediately rinsed in DMEM and lysed with distilled water. The lysate was passed 5X through a 23-gauge needle, plated onto chocolate agar plates in serial dilution, incubated overnight at 37°C and colonies counted to determine NTHi titers. To assess intracellular killing, macrophages were maintained for 3 h after gentamicin removal of extracellular bacteria, and then lysed and the lysates titered. Six wells were used per condition and mouse strain.

### Statistical Analysis

StatView software (version 5.0, JMP-SAS Institute) was used to compare data from WT mice with those from RIP2^−/−^ mice. Two-tailed t-tests with Bonferroni correction were performed for mucosal thickness and ME inflammatory cell numbers (neutrophils and macrophages) at each time point. Two-tailed *t*-tests were also used for macrophage intracellular NTHi titers. The percentages of the ME area covered by leukocytes were compared by the non-parametric Mann-Whitney *U*-test. Differences between the two groups were considered to be significant at *p* < 0.05. The two ears from each mouse were analyzed separately since they were found to be independent from each other (Ebmeyer et al., 2005).

## Results

### RIP2 and Related Genes are Expressed in the ME During OM

**FIGURE 2:**
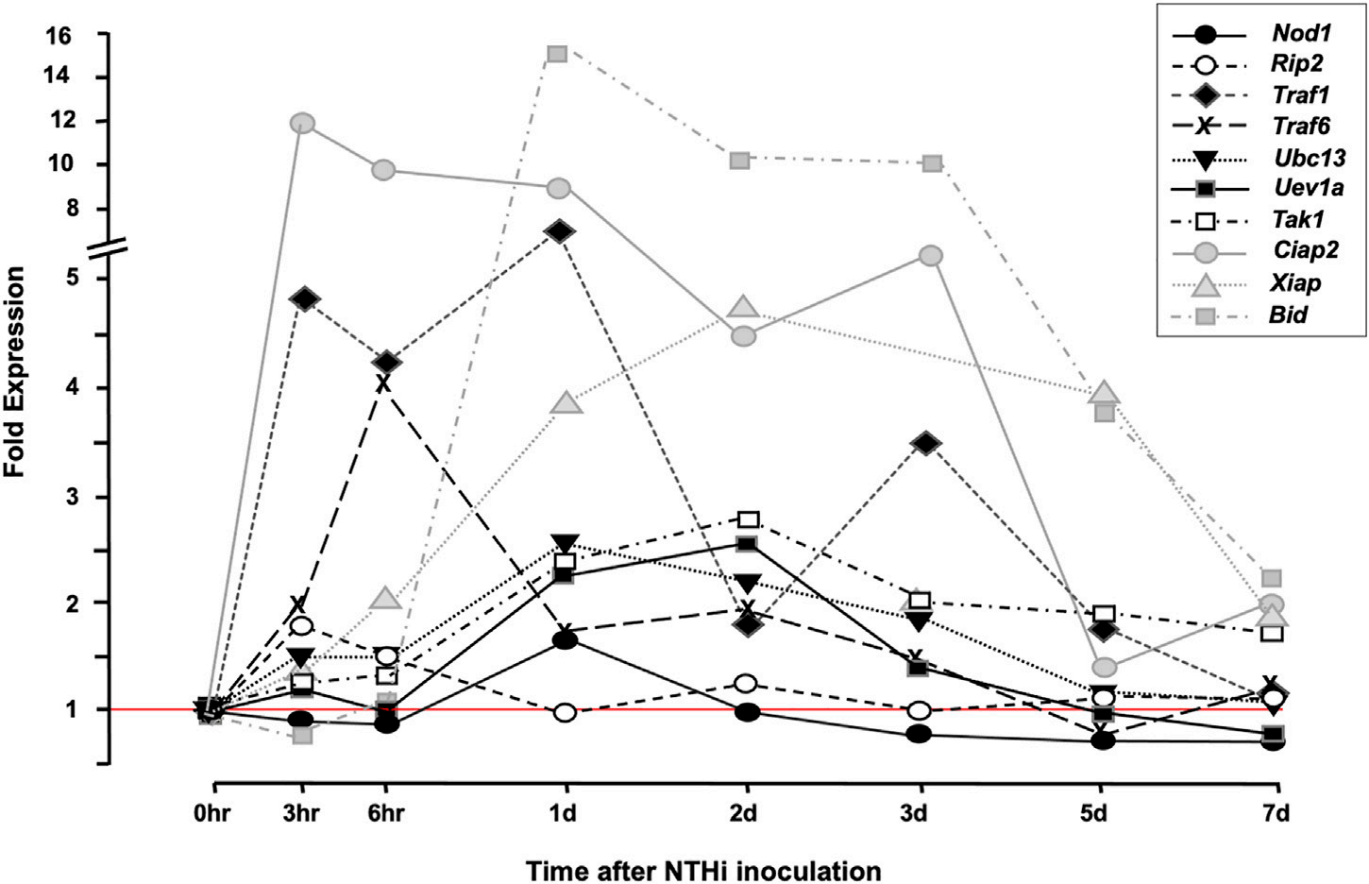
cDNA Array data. Array data from bulk ME RNA provide an overview of gene expression in the ME during OM. The figure presents a comparison of ME gene expression during a complete episode of acute ME infection due to NTHi inoculation, with that observed prior to infection. Many RIP-related genes are up-regulated within hours of ME infection [The data for *Nod1*, *Ripk2*, *Ubc13*, and *Traf6* were published in a prior study (Lee et al., 2019), and are presented here for comparison with other RIP2-related genes].

The gene-array assessment compared the expression of genes in uninfected MEs (0 h) to that observed in MEs 3 h, 6 h, 1, 2, 5, and 7 days after NTHi inoculation, covering an entire episode of acute OM. The array data were mined for RIP2 and related genes that were differentially regulated during OM (Figure 2). Many of the genes for proteins acting up- and down-stream of RIP2 such as *Nod1*, *Uev1a*, *Ubc13*, *Traf1*, *Traf6, Tak1*, and *Bid* were significantly upregulated in WT mouse OM, peaking from 3 h to 1 day after inoculation. *Ripk2* gene itself was triggered as early as 3–6 h post NTHi inoculation. The IAP genes *Ciap1*, *Ciap2*, and *Xiap* were also significantly regulated: *Ciap1* and *Ciap2* within the first few hours and *Xiap* at 2 days. Unfortunately, the *Nod2* gene was not present in the array. Detailed data on fold change ranges and statistics are presented in the Supplementary Table S1.

**FIGURE 3:**
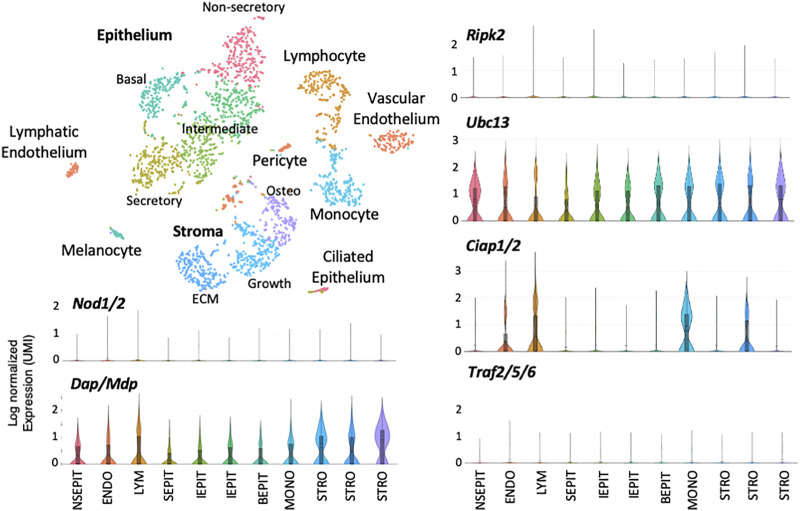
Single-cell RNA-Seq data from the uninfected ME. Single-cell transcriptomes were generated from cells of the normal ME and subjected to PCA to generate clusters based on differential gene expression. Marker genes were used to identify the cell types present in each cluster. Some cell types were divided by PCA into multiple clusters (e.g., epithelial and stromal cells, while others were combined into a single cluster (vascular and lymphatic endothelial cells with pericytes). Expression of genes and gene combinations are presented as violin plots, in which the vertical line represents the highest expressing cell, while the width of the plot represents the number of cells at that normalized log expression level. Notably, the *Nod* and *Ripk2* genes were expressed by very few cells of any type in the normal ME.

**FIGURE 4:**
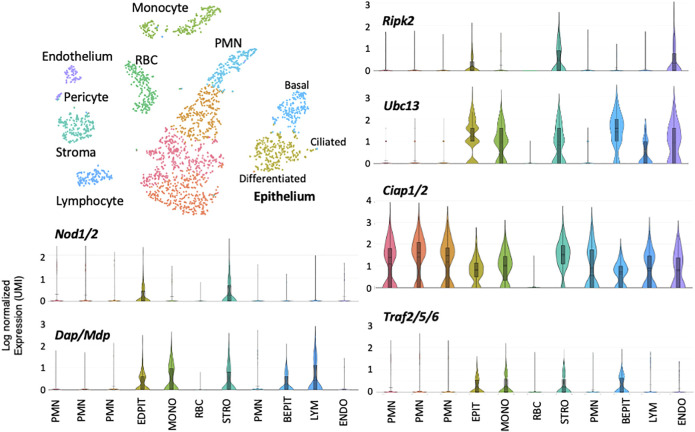
Single-cell RNA-Seq data from the ME 6 h after NTHi infection. Within hours of infection the ME is infiltrated by large numbers of neutrophils (PMNs), and RBCs are also present. Upregulation of many RIP2-related genes among is also evident in some cell types, with the exception of PMNs and RBCs. Expression of the *Nod* genes was prominent only in epithelial and stromal cells. *Ripk2* was similarly observed in epithelial and stromal cells, but in endothelial cells as well. For all other cell types, the great majority of cells showed no expression of these genes. Thus only ME epithelial and stromal cells expressed substantial levels of RIP2-related genes involved in mediating inflammatory and antibacterial responses.

Single-cell RNA-Seq of a normal ME mucosal sample is presented in Figure 3. Eleven cell clusters were generated by PCA. Marker gene expression indicated that five clusters represented epithelial subtypes, three represented stromal subtypes, and individual clusters consisted of monocytes, lymphocytes, and endothelial cells. Violin plots demonstrate that *Nod1*, *Nod2* or *Ripk2* were expressed by very few ME cells of any category, prior to NTHi infection. Some other genes involved in RIP2 signaling were expressed by various cell types, but with very few cells expressing NOD or RIP2, their involvement in RIP2 signaling would be minimal. Figure 4 shows single-cell RNA-Seq results for a ME sample taken 6 h after NTHi inoculation of the ME. PCA generated 11 cell clusters from 2,788 cells, with four representing infiltrating neutrophils (PMNs), two consisting of epithelial cells, and individual clusters of stromal cells, endothelial cells, monocytes, lymphocytes and RBCs. Violin plots of gene expression show increases for *Nods*, *Ripk2*, *Ciap*, and *Traf* genes when compared to the uninfected ME. However, only epithelial and stromal cells showed expression of all elements required for RIP2 signaling.

Since RIP2 may mediate NOD2 autophagy function (Homer et al., 2012; Lee et al., 2019), expression of genes related to autophagy were evaluated before or after ME infection. There were no marked changes noted in the expression of *Becn1*, *Atg5*, *Atg7*, *Atg12*, *Atg16l1* or *Atg16l2* both before and 6 h after infection. As well, these genes were expressed in a small minority of all cell types, except PMNs and RBCs where they were not expressed. Map1lc3a was strongly expressed in epithelial cells, stromal cells, endothelial cells and pericytes, but this did not change with infection.

### RIP2 Deficiency Prolongs ME Inflammation

**FIGURE 5:**
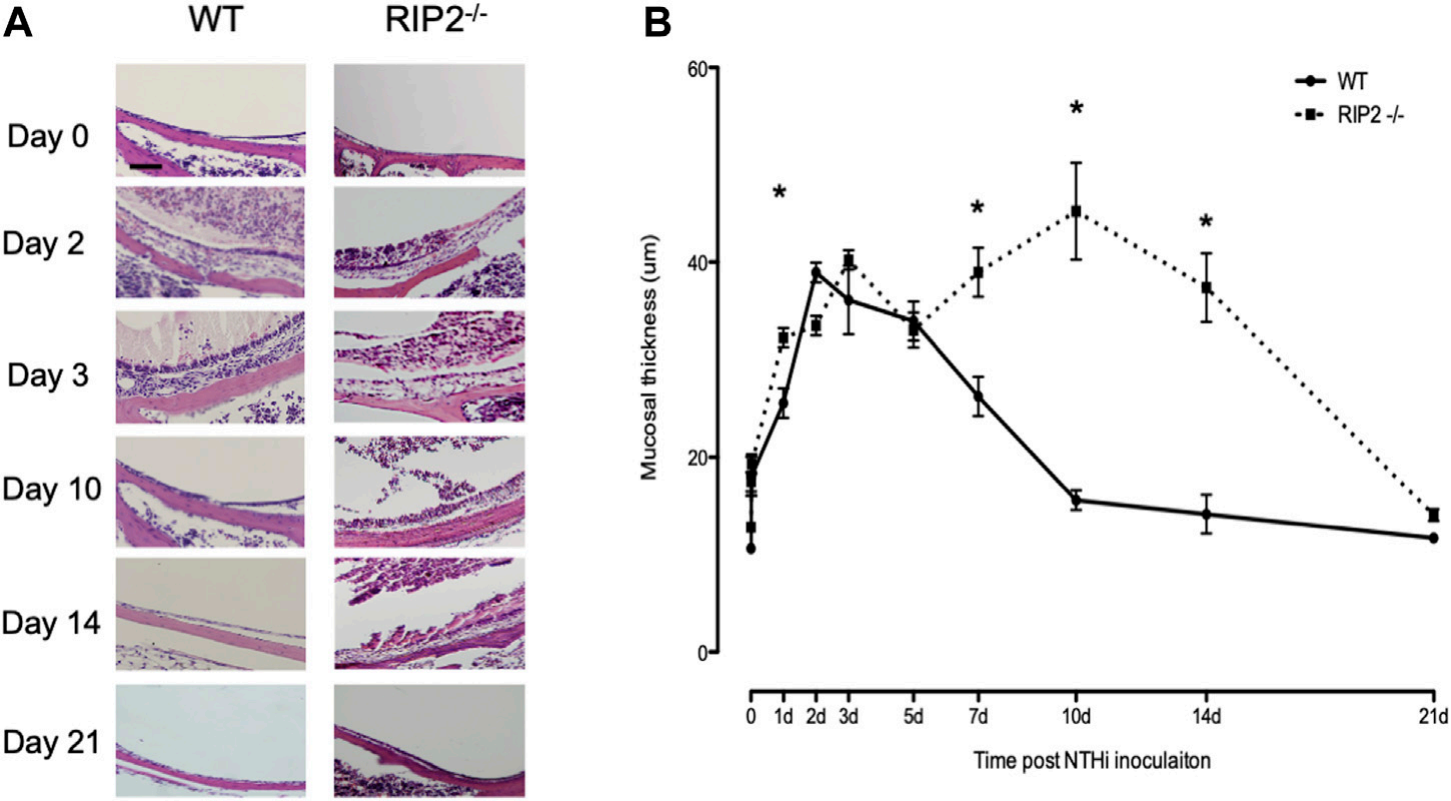
ME response to non-typeable *H. influenzae* (NTHi) in C57BL/6 mice and RIP2^-/-^ mice up to 21 days post inoculation. **(A)** The MEs cavities of C57BL/6 mice were filled with inflammatory cells and transudate fluid at day 2. RIP2^-/-^ mice had fewer inflammatory cells evident in the ME cavity. Starting at day 5, no inflammatory cells were evident in the ME cavity of C57BL/6 mice and the mucosal layer had remodeled to its baseline appearance. Meanwhile, the MEs of RIP2^-/-^ mice showed a persistent inflammatory cell infiltrate and increased mucosal thickness compared with C57BL/6 mice. **(B)** A quantitative evaluation of mucosal thickness of the ME cavity throughout the course of OM. The MEs of C57BL/6 mice and RIP2^-/-^ mice showed similar degrees of mucosal thickness through 12 h after infection with non-typeable *H. influenzae* (NTHi). Thicker mucosal was evident on day 1 for RIP2^-/-^ mice and day 2 for C57BL/6 mice. The degrees of thickness diverged after day 5, when the ME mucosa of RIP2^-/-^ mice kept persistently thick while those of C57BL/6 mice returned to near baseline thickness. Scale bar = 40 μm. All panels are at the same magnification per scale bar provided.

**FIGURE 6:**
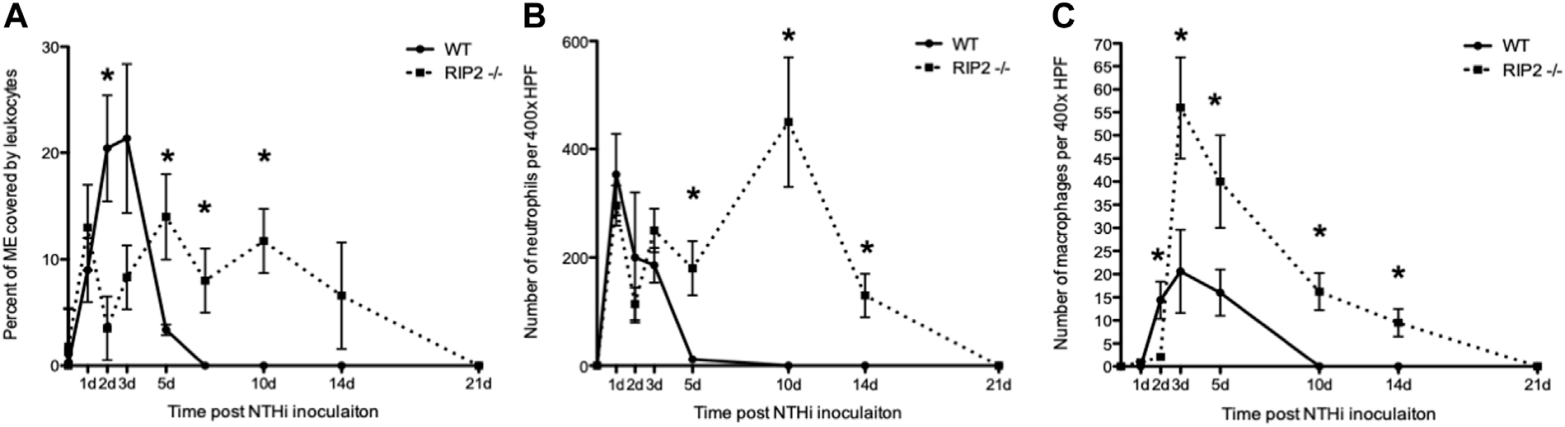
Infiltration of the ME cavity by leukocytes after non-typeable *H. influenzae* inoculation. **(A)** A greater percentage of the ME was occupied by inflammatory cells in C57BL/6 mice than in RIP2^−/−^ mice through 3 days after NTHi infection. Leukocyte infiltration is substantially delayed in RIP2^−/−^ mice, peaking at 5 days after NTHi inoculation, and persisting through day 14 (n = 6–8 MEs per time point; bars represent the mean ±SEM; *p < 0.05. **(B)** Leukocyte numbers for neutrophils measured in ME infiltrates in wild-type and RIP2^−/−^ mice. C57BL/6 mice showed peak neutrophil numbers by day 1 after infection with NTHi with no neutrophils evident by day 10 after infection. Neutrophils showed a striking, late influx after day 5, with the peak at day10 and some neutrophils still present 14 days after infection in RIP2 ^−/−^ mice (n = 6 MEs per time point; bars represent ±SEM; *p < 0.05). **(C)** Numbers for macrophages measured in ME infiltrates in wild-type and RIP2^−/−^ mice. Macrophages were recruited to the ME by 2 days after NTHi infection in C57BL/6 mice, with no macrophages noted by day 10 after infection. The RIP2^−/−^ mice had a short delay in macrophage recruitment by day 2 after infection but displayed prolonged macrophage presence in the ME until 21 days after non-typeable *H. influenzae* (n = 6 MEs per time point; bars represent ±SEM; *p < 0.05).

The normal ME mucosa consists of epithelial and stromal layers, with a sparse vascularization and few resident leukocytes. Normal morphology consists of a monolayer of simple squamous epithelium underlain by a thin stroma with sparse vascularization. The lumen of the ME is also clear of any exudate (Figure 3). During bacterial infection with NTHi, the ME changes dramatically due to the inflammatory response initiated by innate immunity. For C57BL/6 wild-type mice, the height of this inflammation occurs 2–3 days after inoculation, and is reflected histologically by mucosal hyperplasia and leukocyte infiltration of the mucosa and ME cavity. During the first few days of OM, mucosal thickening was comparable for both wild-type and RIP2^−/−^ mice (Figure 5). However, cellular infiltration of the ME lumen of WT mice, was initially lower in RIP2^−/−^ mice (Figures 5, 6). The WT ME mucosa returned to baseline thickness by day 10 after infection. However, mucosal hyperplasia remained high in RIP2^−/−^ mice until 14 days after NTHi infection, recovering to normal by 21 days (Figure 5).

### Delay in Neutrophil and Macrophage Recruitment to the Site of ME Infection in the Absence of Signaling Through RIP2

Infiltration of the ME cavity by leukocytes, as measured by percent of the area of the lumen occupied by cells, peaked at 2–3 days after NTHi inoculation in WT mice, with a rapid decline to normal by 7 days. In RIP2^−/−^ mice infiltration of inflammatory cells was dramatically lower than in the WT on day 2 and 3 after infection, but then rose to a peak at day 5 and remained high until day 14. Clearance of cells did not occur until 21 days after inoculation (Figure 6A).

The relative cellular composition of the ME infiltrate was analyzed by counting neutrophils and macrophages in high-power images of the infiltrate (Figures 6B,C). Neutrophils far outnumbered macrophages the ME cavity at all times. In WT mice, the number of neutrophils peaked at day 1 after NTHi infection and then decreased until few were present on day 5. In RIP2^−/−^ mice, neutrophil counts were similar to WT from day 1 to day 3, but remained high from day 5 to day 14 and only clearing on day 21. Macrophages entered the ME lumen on day 2 in WT mice, remained high through day 5, and were absent by day 10. In RIP2^−/−^ mice, macrophages did not enter the ME until day 3, at which point they peaked. Their numbers then decreased steadily, but did not clear the ME until day 21.

### RIP2 is Required for Normal Bacterial Clearance of the ME

Bacterial clearance after NTHi infection in MEs obtained from C57BL/6 mice was fully resolved by day 5 as no cultures could be seen on plates from that time point forward. However, this recovery was prolonged in RIP2^−/−^ mice until 21 days after infection. NTHi bacteria were able to be successfully retrieved and could be cultured throughout the entire time course. The viable bacteria were gradually cleared from the ME and infection slowly resolved itself, suggesting a defect in the recovery mechanism due to the deletion in the RIP2 gene (Table 1).

**TABLE 1:** Impaired bacterial clearance of RIP2^−/−^ MEs. No colony forming units (CFUs) were detected by day 5 after NTHi inoculation in C57BL/6 mice. Bacterial clearance was impaired in RIP2^−/−^ mice until 21 days after inoculation. NTHi was isolated from more than half of the MEs by 14 days. Mean bacterial colonization of the culture positive plates was evaluated using semi-quantitative analysis of bacterial colonization score (CS): 0 indicated no CFUs, 1 indicates one quadrant with CFUs, 2 indicates two quadrants with CFUs, 3 indicates three quadrants with CFUs and 4 indicates four quadrants with CFUs. Data represent positive culture plates out of 6 MEs.

Time after NTHi inoculation	C57 WT Mean bacterial CS	C57 WT CFU/mL	RIP2^−/−^ Mean bacterial CS	RIP2^−/−^CFU/mL
Day 0	0.00	0.00	0.00	0.00
Day 1	4.00	>10^5^	3.83	>10^5^
Day 2	3.00	>10^4^	4.00	>10^5^
Day 3	1.00	∼500	3.50	>10^4^
Day 5	0.00	0.00	3.20	>10^4^
Day 10	0.00	0.00	3.00	>10^4^
Day 14	0.00	0.00	2.5	∼1000
Day 21	0.00	0.00	0.00	0.00

### Bacterial Phagocytosis and Intracellular Killing is Normal in RIP2-Null Macrophages

**FIGURE 7:**
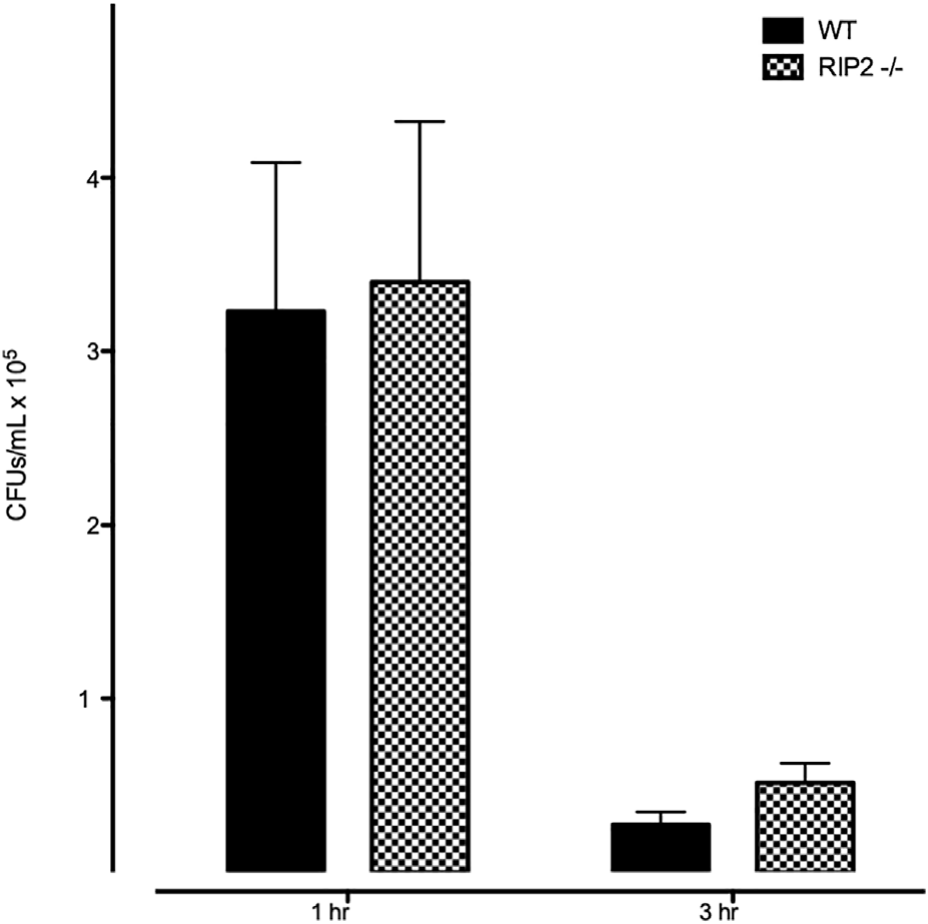
Lack of effect of RIP2 deletion on macrophage NTHi phagocytosis and bacterial killing. In a standard *in vitro* assay for phagocytosis, peritoneal macrophages from WT and RIP2^−/−^ mice exhibited similar uptake of bacteria after 1 h incubation with NTHi, and a similar reduction after 2 additional hours without NTHi exposure.

We evaluated the phagocytic and intracellular killing capacity of peritoneal macrophages derived from RIP2^−/−^ mice *in vitro*. As shown in Figure 7, the number of intracellular bacteria recovered from RIP2^−/−^ macrophages after a 1 h exposure to NTHi is similar to that observed in WT cells. This indicates that macrophage phagocytosis of this bacterial strain is not dependent on RIP2. The number of intracellular bacteria recovered 2 h after the elimination of extracellular NTHi was also similar between WT and RIP2^−/−^ cells, indicating that intercellular killing was unaffected.

## Discussion

We report the first study documenting the role of RIP2 in experimental OM. In a clinical study of PRRs and immunoglobulin gene expression in the peripheral blood leukocytes of OM-prone children, it was found that NOD1 mRNA was down-regulated when compared to OM-normal children (Kaur et al., 2016). Moreover, association studies have linked polymorphisms of various PRRs to OM-proneness in children (Wiertsema et al., 2006; Emonts et al., 2007). While indirect, these human results implicate downstream PRR signaling molecules such as RIP2 as important for the innate immune defense of the ME. A study in mice (Lee et al., 2019) found that deletion of either the NOD1 or NOD2 gene produced a relatively modest prolongation of NTHi-induced OM. Meanwhile, deletion to TLR2 or TLR4 modulated profound prolongation and persistent inflammation up to 21 days (Leichtle et al., 2009). The greater impact of RIP2 could be in part due to its role in the signal amplification cascade and/or activation by other receptors. The present study provides direct support for the hypothesis that RIP2 signaling, both NOD-dependent and independent, is critical for timely OM resolution and the prevention of COM. Of note our gene array date indicate that *Tlr2* is strongly upregulated (Leichtle et al., 2022), which may have a potential role in RIP2 activation (Kobayashi et al., 2002).

While RIP2-mediated responses to infection have been observed in leukocytes at other sites (McCully et al., 2008; Shimada et al., 2018; Honjo et al., 2021), in the ME *Ripk2* and *Nod* gene expression was prominent only in epithelial and stromal cells (Figure 4). These cells are thus the most likely to be contributors to RIP2-mediated responses during OM. In the normal middle ear, we distinguish five known ME epithelial cell types (secretory, non-secretory, intermediate, basal and a small number of ciliated) (Figure 3; Ryan et al., 2020). Gene expression differences between first three of these are not as great as for the last two. However, during OM, when epithelial cells make up much less of the total cell population due to leukocyte influx, PCA clusters the first three categories together as differentiated epithelial cells and generates a separate cluster for basal epithelial cells. Ciliated epithelial cells, typically too few to be distinguished as separate cluster, are included in the differentiated cell cluster, but are always spatially separate (Figure 4).

Mucosal epithelial cells are well known to produce antimicrobial peptides, mucus, cytokines and chemokines upon inflammatory stimulation (Mittal et al., 2014), while stromal cells primarily produce cytokines and chemokines (e.g., Worrell and MacLeod, 2021). Cells from RIP2^−/−^ mice have previously been shown to have disabled production of cytokines such as IL6 and TNFα, normally seen early in an infection, in response to Pathogen-associated molecular patterns (PAMPs) (Kobayashi, et al., 2002). The gene deletion and single-cell gene expression data presented here in combination underscore the importance of cells other than leukocytes in the resolution of OM. Of course, it is possible that additional cell types including leukocytes express RIP2 and NODs at later times after ME infection. However, our gene array data (Figure 2) indicate that RIP2 mRNA is upregulated only at 3 and 6 h after NTHi inoculation, making this unlikely.

Four classes of PMNs were noted after ME infection (Figure 4). Recent research has identified a number of means of classifying neutrophil subpopulations. This includes immature versus mature, N1 (anti-tumorigenic) versus N2 (pro-tumorigenic), tissue injury versus tissue repair, as well as neurorepair subtypes. In other tissues these genes have been distinguished by various patterns of expression, such as EMN positive versus native to distinguish maturity or, Ly6g_lo_ versus Ly6g_hi_, arginase1 positive versus negative, pro-inflammatory versus anti-inflammatory cytokines, or TGFB positive versus negative to distinguish functional subtypes (Kumar et al., 2018; Sas et al., 2020; Fukushima et al., 2021). In the ME we do not see patterns that fit the categories defined in other tissues. In particular, we see no expression of the immature PMN marker *Emn*, although the presence of immature PMNs is virtually certain. Thus while PMNs are functionally complex, but differentiation markers at least in the ME are not yet well defined.

Both C57BL/6 and RIP2^−/−^ mouse MEs are able to recruit leukocytes (Figure 5). However, entry of macrophages into the tympanic cavity of RIP2^−/−^ mice was delayed by 1 day, indicating a corresponding delay in the mechanisms of their recruitment and/or extravasation, such as the expression of macrophage chemotactic chemokines by stromal cells. For example, release of Annexin1 from neutrophils is a powerful macrophage chemoattractant (Kumar et al., 2018). Also, both neutrophils and macrophages persisted in the RIP2^−/−^ ME for a much longer period and in higher numbers than in the WT ME. This persistence seems most likely to reflect failure to clear the ME of NTHi (Table 1), providing a prolonged leukocyte recruitment stimulus independent of RIP2. However, even the larger numbers of leukocytes were unable to resolve OM in a timely manner. While a previous study of RIP2-deficient peritoneal macrophages showed reduced intracellular killing of *Staphylococcus aureus* (McCully et al., 2008), we did not see a defect in either NTHi phagocytosis or killing by RIP2^−/−^ macrophages (Figure 7). At least for macrophages, defects in their ability to clear bacteria could not explain the deficit in OM resolution. Since RIP2 and NOD gene expression was not prominent in leukocytes in the ME, either before or after NTHi inoculation (Figures 3, 4), this is not surprising.

Apoptosis is an important effector mechanism for the removal of intracellular bacterial replication niches within the host. RIP2 interacts with members of the TRAF (TNFR-associated factor) as well as IAP (inhibitor of apoptosis protein) families, independent of the CARD domain that mediates NOD interactions. TRAFs and IAPs play prominent roles in regulating programmed cell death. Their ability to bind to and reduce the activation of caspases is enhanced by RIP2 (Bertrand et al., 2009; Krieg et al., 2009). RIP2, which mediates the apoptosis of cells with intracellular pathogen infection (Humphries et al., 2015) could cause apoptosis of ME epithelial or stromal cells infected with bacteria. It can be speculated that its lack results in persistent intracellular reservoirs of NTHi and prolongs the presence of bacteria in the ME.

Although NTHi was cleared from the ME cavity of all RIP2-null mice by 21 days after inoculation, many were able to eliminate bacteria after only 14 days. This suggests that there are alternative pathways that can mediate anti-bacterial responses besides those involving RIP2. Prime candidates are the TLRs, activated by many of the same PAMPs as the NODs and also responding to lipopolysaccharides. While TLRs have been reported to activate RIP2 (Kobayashi et al., 2002), the primary downstream pathway of most TLRs is via the adaptor molecule MyD88 (Kawai and Akira 2011). We previously found that deletion of MyD88 in mice produced a delay in the resolution of NTHi-induced OM that is similar to that induced by lack of RIP2 (Hernandez et al., 2008). In addition, the NLR NALP3 (or cryopyrin) acts independently of RIP2. It interacts with the CARD-containing adaptor molecule ASC and caspase 1 to form the inflammasome, which is responsible for the maturation of IL-1beta and IL-18 as well as promotion of cell death. It has been associated with inflammatory diseases such as familial cold-induced autoinflammatory syndrome and neonatal-onset multisystem inflammatory disease, which group together to form the cryopyrin-associated periodic syndromes (Walsh, 2009). Intracellular receptors that can be activated by bacterial DNA (Leichtle et al., 2012) are another RIP2-independent source of innate immune activation.

Given the existence of several alternative innate immune pathways, it is perhaps surprising that the elimination of RIP2 has such a profound effect on OM pathogenesis and recovery. We have previously noted that the individual elimination of most innate immune molecules will disable OM recovery to a greater or lesser degree (Kurabi et al., 2016). This suggests that virtually all innate immune pathways contribute to the defense of the ME from bacterial infection, and that they must act in concert to orchestrate timely recovery from OM. RIP2 appears to induce host resistance at the early stages of OM infection since RIP2 deficiency resulted in prolonged bacterial loads (to at least 14 days) and persistent OM pathogenesis.

RIP2 is recognized an important mediator of inflammatory responses, and RIP2 inhibitors have been proposed for the treatment of inflammatory diseases (e.g., Jun et al., 2013). However, we saw no evidence that lack of RIP2 reduced either mucosal hyperplasia or, beyond a temporary reduction in PMN entry and a slight delay in macrophage entry, the infiltration of inflammatory leukocytes into the ME. Especially given its critical role in recovery of the ME from infection, RIP2 inhibitors would likely not be useful as OM therapies.

## Data Availability

The data presented in the study are deposited in the Science Data Bank repository, https://www.scidb.cn/en/s/nuIRzq, accession number 31253.11.sciencedb.01860.
