# Supplementary material for: Essential Role of the Innate Immune Adaptor RIP2 in the Response to Otitis Media
Source: Front Genet. 2022 Jul 12;13:893085. doi: 10.3389/fgene.2022.893085 (PMC9315102; doi:10.3389/fgene.2022.893085)
Supplement: Supplementary file 1 [file Table1.pdf]

Supplementary Table 1. RIP2 pathway signaling related genes fold-change during acute OM: median (range) and *p* value. These values are graphed in Figure 2.

Time	Fold change (range)	P value
***Nod1* 1454733_at**		
Time 0h	1.000 (0.997 to 1.003)	0.999
Time 3h	0.896 (0.815 to 0.922)	0.261
Time 6h	0.738 (0.696 to 0.782)	0.120
**Time 1d**	**1.680 (1.661 to 1.699)**	**0.014**
Time 2d	0.969 (0.891 to 1.053)	0.777
Time 3d	0.658 (0.596 to 0.728)	0.150
Time 5d	0.549 (0.503 to 0.600)	0.093
Time 7d	0.537 (0.533 to 0.541)	0.081
Nod2 (not on the array)		
***Rip2* (*Ripk2*) 1421236_at**		
Time 0h	0.998 (0.930 to 1.070)	0.978
Time 3h	1.777 (1.412 to 2.236)	0.242
**Time 6h**	**1.500 (1.489 to 1.512)**	**0.012**
Time 1d	0.920 (0.800 to 1.058)	0.658
**Time 2d**	**1.235 (1.219 to 1.251)**	**0.040**
Time 3d	0.998 (0.847 to 1.177)	0.994
Time 5d	1.143 (1.085 to 1.205)	0.237
Time 7d	1.137 (1.049 to 1.233)	0.358
***Traf1* 1423602_at**		
Time 0h	0.96 (0.722 to 1.278)	0.911
**Time 3h**	**4.718 (4.42 to 5.037)**	**0.027**
**Time 6h**	**4.22 (4.17 to 4.271)**	**0.005**
**Time 1d**	**7.627 (6.895 to 8.435)**	**0.032**
Time 2d	1.644 (0.9 to 3.004)	0.561
**Time 3d**	**3.51 (3.212 to 3.835)**	**0.045**
Time 5d	1.783 (1.659 to 1.916)	0.079
Time 7d	1.198 (0.942 to 1.522)	0.59
***Traf5* 1447682_x_at** (NOT REGULATED)		
Time 0h	0.994 (0.888 to 1.112)	0.964
Time 3h	0.297 (0.233 to 0.378)	0.125
Time 6h	0.885 (0.84 to 0.933)	0.258
Time 1d	0.645 (0.314 to 1.323)	0.651
Time 2d	0.0817 (0.0495 to 0.135)	0.126
Time 3d	1.094 (0.952 to 1.257)	0.636
Time 5d	0.776 (0.641 to 0.939)	0.41
Time 7d	1.061 (0.734 to 1.533)	0.899
***Traf6* 1435350_at**		
Time 0h	0.964 (0.732 to 1.268)	0.914
**Time 3h**	**2.015 (1.942 to 2.091)**	**0.034**
Time 6h	4.082 (2.916 to 5.716)	0.150
Time 1d	1.596 (1.476 to 1.725)	0.105
Time 2d	1.815 (1.683 to 1.957)	0.0803
Time 3d	1.485 (1.235 to 1.785)	0.278
**Time 5d**	**0.576 (0.563 to 0.589)**	**0.026**
**Time 7d**	**1.218 (1.217 to 1.218)**	**0.001**

***Ubc13* (*Ube2n*) 1422559_at**

Time 0h	0.980 (0.800 to 1.200)	0.936
Time 3h	1.525 (1.424 to 1.633)	0.102
Time 6h	1.528 (1.385 to 1.687)	0.145
**Time 1d**	**2.586 (2.503 to 2.671)**	**0.022**
**Time 2d**	**2.212 (2.120 to 2.308)**	**0.034**
**Time 3d**	**1.737 (1.707 to 1.768)**	**0.020**
Time 5d	1.186 (1.159 to 1.214)	0.087
Time 7d	1.087 (1.017 to 1.162)	0.428

***Uev1A (Ube2v1)* 1444523_s_at**

Time 0h	0.987 (0.839 to 1.161)	0.949
**Time 3h**	**1.171 (1.17 to 1.172)**	**0.003**
Time 6h	1.028 (0.919 to 1.151)	0.847
Time 1d	2.269 (2.083 to 2.473)	0.066
Time 2d	2.546 (2.288 to 2.833)	0.072
Time 3d	1.44 (1.117 to 1.855)	0.387
Time 5d	0.922 (0.906 to 0.939)	0.140
Time 7d	0.769 (0.721 to 0.82)	0.153

***Tak1* (*Map3k7*) 1425795_a_at**

Time 0h	0.997 (0.929 to 1.071)	0.977
Time 3h	1.165 (1.109 to 1.223)	0.198
**Time 6h**	**1.282 (1.272 to 1.291)**	**0.019**
Time 1d	2.353 (2.083 to 2.657)	0.090
Time 2d	2.743 (2.325 to 3.237)	0.104
Time 3d	2.082 (1.927 to 2.251)	0.067
**Time 5d**	**1.801 (1.749 to 1.855)**	**0.032**
Time 7d	1.750 (1.661 to 1.845)	0.060

***Tab1* 1426898_at**

Time 0h	1.000 (0.975 to 1.025)	0.992
Time 3h	0.484 (0.450 to 0.521)	0.064
Time 6h	0.343 (0.248 to 0.475)	0.188
Time 1d	0.765 (0.680 to 0.860)	0.263
Time 2d	0.586 (0.535 to 0.641)	0.107
Time 3d	0.667 (0.577 to 0.771)	0.218
**Time 5d**	**0.671 (0.655 to 0.688)**	**0.040**
Time 7d	0.902 (0.871 to 0.934)	0.209

***Tab2* 1451003_at**

Time 0h	0.999 (0.948 to 1.052)	0.983
Time 3h	1.119 (1.037 to 1.208)	0.378
Time 6h	1.203 (1.102 to 1.312)	0.281
**Time 1d**	**2.16 (2.109 to 2.212)**	**0.020**
Time 2d	2.076 (1.958 to 2.202)	0.051
**Time 3d**	**1.775 (1.735 to 1.815)**	**0.025**
Time 5d	1.254 (1.219 to 1.29)	0.080
**Time 7d**	**1.317 (1.29 to 1.345)**	**0.049**

***Tab3* 1428762_at**

Time 0h	0.985 (0.827 to 1.173)	0.945
Time 3h	1.086 (0.99 to 1.191)	0.535
Time 6h	1.278 (0.907 to 1.799)	0.604
**Time 1d**	**1.343 (1.329 to 1.357)**	**0.023**
Time 2d	1.878 (1.249 to 2.823)	0.366
Time 3d	1.559 (1.284 to 1.893)	0.262
**Time 5d**	**1.086 (1.085 to 1.087)**	**0.008**
Time 7d	1.103 (0.971 to 1.253)	0.582

***Ciap1* 1418854_at** (NOT REGULATED)

Time 0h	1 (0.972 to 1.028)	0.991
Time 3h	2.631 (2.395 to 2.891)	0.062
Time 6h	2.828 (2.436 to 3.284)	0.091
Time 1d	1.111 (1.041 to 1.187)	0.354
Time 2d	1.059 (1.038 to 1.08)	0.210
Time 3d	1.368 (1.192 to 1.571)	0.264
Time 5d	1.172 (1.146 to 1.199)	0.090
Time 7d	1.189 (1.143 to 1.236)	0.141

***Ciap2* 1421392_a_at**

Time 0h	0.997 (0.924 to 1.076)	0.976
**Time 3h**	**11.9 (10.27 to 13.8)**	**0.038**
Time 6h	9.944 (7.701 to 12.84)	0.070
**Time 1d**	**9.764 (8.393 to 11.36)**	**0.042**
Time 2d	4.524 (3.637 to 5.628)	0.091
**Time 3d**	**5.567 (5.56 to 5.575)**	**0.0005**
Time 5d	1.417 (1.241 to 1.618)	0.231
**Time 7d**	**2.205 (2.096 to 2.32)**	**0.041**

***Xiap* 1450231_a_at**

Time 0h	0.999 (0.965 to 1.035)	0.989
Time 3h	1.395 (1.233 to 1.579)	0.226
**Time 6h**	**2.126 (2.057 to 2.197)**	**0.028**
**Time 1d**	**3.803 (3.466 to 4.173)**	**0.044**
Time 2d	4.732 (4.17 to 5.37)	0.052
Time 3d	2.086 (1.06 to 4.107)	0.474
Time 5d	4.033 (3 to 5.422)	0.133
Time 7d	2.043 (1.826 to 2.286)	0.099

***Bid* 1448560_at**

Time 0h	0.99 (0.858 to 1.142)	0.955
Time 3h	0.67 (0.6 to 0.749)	0.173
Time 6h	1.07 (0.524 to 2.187)	0.94
**Time 1d**	**15.39 (14.09 to 16.81)**	**0.021**
Time 2d	9.941 (7.288 to 13.56)	0.085
Time 3d	10.16 (7.601 to 13.57)	0.079
Time 5d	3.809 (2.975 to 4.877)	0.116
Time 7d	2.326 (0.703 to 7.694)	0.609
